# Does early initiation of ART in infants affect virological and resistance outcomes? Data from the CHER trial after 6 years of follow-up

**DOI:** 10.7448/IAS.15.6.18085

**Published:** 2012-11-11

**Authors:** A Violari, M Cotton, K Otwombe, G Hunt, M Kalimashe, R Panchia, L Morris, D Pillay, A Babiker, D Gibb

**Affiliations:** 1 University of the Witwatersrand, Perinatal HIV Research Unit, Johannesburg, South Africa; 2 Stellenbosch University, Children's Infectious Diseases Clinical Research Unit, Tygerberg, South Africa; 3 National Institute for Communicable Diseases (NICD), AIDS Virus Research Unit, Johannesburg, South Africa; 4 University College London, London, UK; 5 MRC Clinical Trials Unit, London, UK

## Abstract

**Purpose of the study:**

Virological outcomes and resistance patterns in children initiating protease inhibitor (PI)-based antiretroviral therapy (ART) immediately following HIV-1 diagnosis are not well described. Challenges include maintaining adherence in asymptomatic patients with very high pre-ART viral loads. The CHER trial compared deferred but continuous ART (arm 1) with early limited ART (arms 2 and 3).

**Methods:**

LPV/r+ZDV+3TC was commenced either immediately (in 250 of 252 children randomized in arms 2 and 3) or at clinical/immunological progression (103 of 125 children in arm 1). Interruption of ART occurred after 40 (arm 2) or 96 weeks (arm 3) and re-initiation with LPV/r + ZDV + 3TC was based on immunologic/clinical criteria. Viral load was measured on all children with a stored specimen at their last visit, having been on initial or restarted ART following interruption (arms 2 and 3) for at least 24 weeks. Children in arms 1, 2 and 3 not initiating ART due to death (16, 0, 0), LTFU (2, 2, 0) or other reason (4, 0, 0) are excluded. Resistance testing was performed on samples with a viral load (VL) ≥1000 c/mL together with the matched baseline sample, if available. Reverse transcriptase (NRTI and NNRTI) and PI inhibitor mutations were analyzed using a validated in-house population-based sequencing assay and the IAS 2011 mutation list.

**Summary of results:**

A total of 377 infants were enrolled; median was age 7.4 (interquartile range (IQR) 6.7 to 8.9) weeks and median baseline viral load was log_10_ 5.7. By end of study (June 2011), 353/377 children had started LPV/r + ZDV + 3TC. Median (IQR) age at ART initiation in arms 1, 2 and 3 was 26.1 (19.9 to 40), 7.4 (6.6 to 8.7) and 7.5 (6.6 to 9.0) weeks. Median (IQR) duration on ART was 240 (216 to 252), 243 (200 to 260) and 240 (194 to 257) weeks in arms 1, 2 and 3, respectively. HIV-1 RNA was <400 c/mL in 88/101 (87%), 95/113 (84%) and 97/117 (83%) (*P*=0.96). Twenty-two of thirty-two children with VL >1000 c/mL (2/5, 8/14, 12/13 in arms 1, 2 and 3) have had resistance tests to date; nine (41%) had mutations. There were seven with M184V mutations (1, 4, 2 in arms 1, 2 and 3); two with major PI mutations (V82A/L76V) (one in each of arms 1 and 2); and two with major NNRTI mutations (K103N/M230L) (one in each of arms 2 and 3). Two of ten children tested to date had NNRTI mutations prior to starting PI-based triple therapy.

**Conclusions:**

Virological response on ART was excellent in this large cohort of infants initiating LPV/r+ZDV+3TC at a very young age, with no differences between randomized strategies, suggesting that planned interruption after early limited ART does not adversely affect virological outcomes. Overall, approximately 40% of those on ART with VL>1000 c/mL had a resistance mutation; PI mutations were infrequent, despite around 5 years on therapy. Ongoing work will investigate impact of length of time with detectable viral load on risk of developing resistance.

